# Supplementary material for: Breaking the mold: telescoping drives the evolution of more integrated and heterogeneous skulls in cetaceans
Source: PeerJ. 2022 May 5;10:e13392. doi: 10.7717/peerj.13392 (PMC9080436; doi:10.7717/peerj.13392)
Supplement: Supplemental Information 3 [file peerj-10-13392-s003.docx]

**Supplemental Tables**

**Breaking the mold: telescoping drives the evolution of more integrated and heterogeneous skulls in cetaceans**

Mónica Romina Buono & Evangelos Vlachos

**Table S1**: List of the specimens used for the construction of the anatomical networks of the skulls and the sources. Physical maturity of the specimens was taken from the literature or determined following the criteria of closure of the cranial sutures suggested by Perrin (1975) and Walsh & Berta (2011) unless indicated.

| **TAXA** | **Family** | **Taxon** | **Collection number** | **Physical maturity** | **Reference** |
| --- | --- | --- | --- | --- | --- |
| **STEM CETACEA** | Pakicetidae | *Pakicetus attocki*  *Pakicetus inachus* | H-GSP 18467  18470,  96231,  96623  GSP-UM084 | -  -  adult  juvenile  adult^1^ | Nummela et al., 2006  Gingerich & Russell, 1981; Gingerich & Russel 1990 (Fig. 8); |
|  | “Protocetidae” | *Aegyptocetus*  *Georgiacetus*  Protocetidae indet.  *Artiocetus clavis* | MSNTUP I-15459  GSM350  CMM-V-4536  GSP-UM3458 | adult    adult  -  - | Bianucci &  Gingerich, 2011  Hulbert et al., 1998  Godfrey et al., 2013  Gingerich et al., 2001 |
|  | Basilosauridae | *Dorudon atrox* | UM 100139  UM 93220  UM 101222  UM 97512  UM 97506 | juvenile  juvenile  adult  adult  adult | Uhen, 2004;  Marx et al 2016 (fig. 3.8) |
| **MYSTICETI** | Aetiocetidae | *Aetiocetus cotylalveus*  *Aetiocetus*  *weltoni* | USNM 25210  UCMP12290 | adult  subadult | Emlong, 1966  Demeré & Berta, 2008  Marx et al 2016 (Fig. 4.10) |
|  | Eomysticetidae | *Yamatocetus* | KMNH VP 000,017 | subadult^2^ | Okazaki 2012 (p:4);  Marx et al. 2016 fig. 4.12 |
|  | Balaenidae | *Eubalaena australis* | CNP-MAMM  748  MLP 1508  MoNZ 2239  USNM 267612 | neonate  subadult  juvenile?^3^  adult | Marx et al., 2016  Tsai et al., 2014  MB personal observations |
|  | Balaenopteridae | *Balaenoptera acutorostrata*  *B.borealis*  *B. physalus*  *B. musculus*  *B. edeni*  *B.omurai*  *B.brydei*  *B. ricei* | CNP-MAMM 100750  USNM 61715  USNM 504244  USNM 236680  USNM 550467  USNM 16039  USNM 124326  GRM223  NSMT-M32505  RMNH 78N33  USNM 594665 | juvenile  subadult  subadult  subadult  subdult  adult  adult  adult  adult  adult  adult | Marx et al., 2016 (fig. 4.17)  Muller, 1954; Ichishima, 2016  Tsai et al., 2014  Wada et al., 2003 (fig.1)  Yamada et al 2006  Tsai et al., 2014  Rosel et al., 2021 |
|  | Cetotheriidae | *Caperea* | NMNZ MM  2235  NMNZ MM  2254 | adult^5^  subadult^5^ | Marx et al., 2016  Tsai et al., 2014 |
|  |  | *Piscobalaena nana* | MNHN SAS 1617  MNHN SAS 1618  MNHN SAS 1623 | adult  adult  adult | Bouetel et al., 2006 |
| **ODONTOCETI** | Xenorophidae | *Albertocetus meffordorum* | CCNHM 303  CCNHM 218  USNM 525001 | subadult  adult  subadult | Boessenecker et al., 2017;  Uhen, 2008 |
|  | Platanistoidea | *Waipatia* | OU 22095 | subadult/  adult | Fordyce, 1994 |
|  | Platanistoidea | *Notocetus vanbenedeni* | MLP 5-5,  5-10 | adult  adult | Viglino, 2019 personal communications from M.Viglino (August, 2021) |
|  | Physeteridae | *Physeter* | USNM 35315    MACN-Ma 29768 | subadult  adult | Flower,1868^4^  personal observations from F. Paolucci, (June 2021) |
|  | Delphinidae | *Tursiops truncatus* | USNM 550403  CNP-MAM 100-644  CNP-MAM 100953  CNP-MAM 100952  CNP-MAM 100954  CNP-MAM 100957 | adult  adult  subadult  adult  adult  adult | Mead & Fordyce (2009) |

^1^ Based on a byzigomatic width of 145mm (Gingerich & Russel, 1981), higher than the adult specimen H-GSP 96231 of *Pakicetus attocki.* There is not evidence in the dental morphology (e.g. deciduous dentition) or cranial futures suggesting a juvenile condition.

^2^ Based on the degree of cranial close suture and fusion of vertebral and forelimb epiphysis

^3^ Specimen with basioccipital-basisphenoid suture completely closed; however, most of the basicranial sutures (e.g. squamosal-alisphenoid; pterygoid-basioccipital; pterygoid/palatine) are not fused which might indicate a juvenile condition. Besides, cancellous bones surface of squamosal/pterygoid also indicates an immature condition.

^4^ This work includes an adult specimen and comparison with a juvenile.

^5^ Based on information available in Buchholtz (2011)

**Institutional abbreviations**: **CCNHM**, Mace Brown Museum of Natural History, Charleston, South Carolina, USA; **CMM-V**, Calvert Marine Museum fossil Vertebrate collection, Solomons, Maryland, USA; **CNP-MAMM**, Laboratorio de Mamíferos Marinos, Centro Nacional Patagónico, Puerto Madryn, Argentina; **GSP-UM**, Geological Survey of Pakistan, University of Michigan; Michigan, USA; **GRM**, Indian Museum, Kolkata, Indian; **GSM**, Georgia Southern Museum, Statesboro, Georgia, USA; **H-GSP**, Geological Survey of Pakistan, Howard University, Washington, D.C., USA; **KMNH**, Kitakyushu Museum of Natural History and Human History, Kitakyushu, Japan; **MLP**, Museo de La Plata, La Plata, Argentina; **MNHN**, Museum National d`Histoire Naturelle, Paris, France; **NMNZ**, Museum of New Zealand Te Papa Tongarewa, Wellington, New Zealand, **NSMT-M,** National Science Museum, Tokyo, Japan; **OU**, Geology Museum, University of Otago, Dunedin, New Zealand; **RMNH,** National Museum of Natural History, Leiden; **UCMP**, University of California Museum of Paleontology, Berkeley, USA; **UM**, University of Michigan Museum of Paleontology, Ann Arbor, Michigan; **USNM**, National Museum of Natural History, Washington, D.C., USA;

**Table S2.** PERMANOVA analysis considering the taxonomic classification of the tetrapods groups. Statistically significant p < 0.05 marked with bold.

| p/F | Ichthyostegalia | Seymouriamorpha | Amphibia | Procolophonomorpha | Testudinata | Sauropsida | Crocodilia | Dinosauria | Aves | Lepidosauria | Squamata | Synapsida | Mammalia | Cetacea |
| --- | --- | --- | --- | --- | --- | --- | --- | --- | --- | --- | --- | --- | --- | --- |
| Ichthyostegalia |  | 1 | 0.4967 | 1 | 0.1092 | 1 | 1 | 0.7994 | 1 | 1 | 0.5162 | 0.8085 | 0.1267 | **0.0441** |
| Seymouriamorpha | 12.39 |  | 0.2498 | 1 | 0.104 | 1 | 1 | 0.7921 | 1 | 1 | 0.5063 | 0.1989 | 0.1263 | **0.0452** |
| Amphibia | 2.37 | 2.86 |  | 0.5026 | 0.0824 | 0.1008 | 0.7564 | **0.0324** | 0.7533 | 0.5013 | **0.0333** | **0.0327** | 0.2638 | 0.087 |
| Procolophonomorpha | 2.86 | 2.86 | 0.9135 |  | 0.1037 | 0.2461 | 1 | **0.5917** | 1 | 1 | 0.7559 | 0.605 | 0.1216 | **0.0442** |
| Testudinata | 17.74 | 23.12 | 3.179 | 3.691 |  | **0.0049** | 0.3976 | 0.0034 | 0.1018 | 0.1015 | **0.0039** | **0.0013** | **0.0002** | **0.0001** |
| Sauropsida | 0.05763 | 0.106 | 8.539 | 1.781 | 35.18 |  | 0.2506 | 0.3193 | 0.243 | 1 | 0.2077 | 0.1721 | **0.0092** | **0.0004** |
| Crocodilia | 35.18 | 35.18 | 0.7009 | 0.7009 | 1.865 | 2.736 |  | 0.5975 | 1 | 1 | 0.6226 | 0.206 | 0.1203 | **0.045** |
| Dinosauria | 0.2654 | 0.5267 | 6.728 | 0.5874 | 16.93 | 1.203 | 0.8705 |  | 0.1979 | 0.8 | 0.4592 | 0.4014 | **0.004** | **0.0002** |
| Aves | 0.8705 | 0.8705 | 0.2868 | 0.2868 | 12.66 | 13.38 | 13.38 | 8.477 |  | 1 | 0.1258 | 0.203 | 0.3738 | **0.0428** |
| Lepidosauria | 8.477 | 8.477 | 1.954 | 1.954 | 13.05 | 0.5914 | 0.5914 | 0.1744 | 0.1744 |  | 0.6271 | 0.8031 | 0.1291 | **0.0476** |
| Squamata | 0.4184 | 0.8273 | 5.813 | 0.09684 | 8.598 | 1.726 | 0.3968 | 0.6537 | 4.653 | 0.2977 |  | 0.8426 | **0.0009** | **0.0001** |
| Synapsida | 0.8627 | 2.086 | 7.563 | 0.8387 | 24.96 | 2.051 | 2.329 | 0.9133 | 19.41 | 0.8431 | 0.1547 |  | 0.0034 | **0.0002** |
| Mammalia | 18.15 | 20.94 | 1.496 | 9.211 | 34.23 | 48.01 | 7.649 | 38.97 | 1.492 | 15.58 | 32.49 | 47.27 |  | **0.0001** |
| Cetacea | 29.04 | 37.17 | 3.224 | 8.692 | 16.73 | 73.35 | 7.935 | 50.47 | 6.701 | 22.76 | 29.96 | 53.08 | 41.55 |  |

**References**

Bianucci, G, & Gingerich, PD. (2011). *Aegyptocetus tarfa*, n. gen. et sp. (Mammalia, Cetacea), from the middle Eocene of Egypt: clinorhynchy, olfaction, and hearing in a protocetid whale. *Journal of Vertebrate Paleontology*, 31: 1173-1188.

Boessenecker, RW, Ahmed, E, & Geisler, JH. (2017). New records of the dolphin *Albertocetus meffordorum* (Odontoceti: Xenorophidae) from the lower Oligocene of South Carolina: encephalization, sensory anatomy, postcranial morphology, and ontogeny of early odontocetes. *PLoS One*, 12: e0186476.

Bouetel, V, & de Muizon, C. (2006). The anatomy and relationships of *Piscobalaena nana* (Cetacea, Mysticeti), a Cetotheriidae ss from the early Pliocene of Peru. *Geodiversitas*, 28: 319-395.

Buchholtz, EA. (2011). Vertebral and rib anatomy in *Caperea marginata*: implications for evolutionary patterning of the mammalian vertebral column. *Marine Mammal Science*, 27: 382-397.

Deméré, TA, & Berta, A. (2008). Cranial anatomy of the toothed mysticete *Aetiocetus weltoni* and its implications for aetiocetid phylogeny. *Zoological Journal of the Linnean Society*, 154: 308-352.

Emlong, DR. (1966). A new archaic cetacean from the Oligocene of northwest Oregon. *Bulletin of the Museum of Natural History*, University of Oregon 3: 1-51.

Flower, WH. (1868). On the osteology of the cachalot or sperm-whale *(Physeter macrocephalus). Transactions of the Zoological Society of London* 6: 309-372.

Fordyce, RE, & Barnes, LG. (1994). The evolutionary history of whales and dolphins. *Annual Review of Earth and Planetary Sciences*, 22: 419-455.

Gingerich, PD, & Russell, DE. (1981). *Pakicetus inachus*, a new archaeocete (Mammalia, Cetacea) from the early-middle Eocene Kuldana Formation of Kohat (Pakistan). *Contributions from the Museum of Paleontology the University of Michigan*, 25: 235-246.

Gingerich, PD, & Russell, DE. (1990). Dentition of early Eocene *Pakicetus* (Mammalia, Cetacea). *Contributions from the Museum of Paleontology the University of Michigan*, 28:1-20.

Gingerich, PD, ul Haq, M., Zalmout, IS, Khan, IH, & Malkani, MS. (2001). Origin of whales from early artiodactyls: hands and feet of Eocene Protocetidae from Pakistan. *Science*, 293: 2239-2242.

Godfrey, SJ, Geisler, J, & Fitzgerald, EM. (2013). On the olfactory anatomy in an archaic whale (Protocetidae, Cetacea) and the minke whale *Balaenoptera acutorostrata* (Balaenopteridae, Cetacea). *The Anatomical Record*, 296: 257-272.

Hulbert, RC, Petkewich, RM, Bishop, GA, Bukry, D, & Aleshire, DP. (1998). A new middle Eocene protocetid whale (Mammalia: Cetacea: Archaeoceti) and associated biota from Georgia. *Journal of Paleontology*, 72: 907-927.

Ichishima, H. (2016). The ethmoid and presphenoid of cetaceans. *Journal of Morphology*, 277: 1661-1674.

Marx, FG, Lambert, O, & Uhen, MD. (2016). Cetacean paleobiology. West Sussex: John Wiley & Sons.

Mead JG, Fordyce RE. 2009. The therian skull: a lexicon with emphasis on the odontocetes. *Smithsonian Contributions to Zoology,* 627:1-261.

Muller, J. (1954). Observations on the orbital region of the skull of the Mystacoceti. *Zoologische Mededelingen*, 32: 279-290.

Nummela, S, Hussain, ST, & Thewissen, JGM. (2006). Cranial anatomy of Pakicetidae (Cetacea, Mammalia). *Journal of Vertebrate Paleontology*, 26: 746-759.

Okazaki, Y. (2012). A new mysticete from the upper Oligocene Ashiya Group, Kyushu, Japan and its significance to mysticete evolution. *Bulletin of the Kitakyushu Museum of Natural History and Human History,* Series A (Natural History), 10: 129-152.

Rosel, PE, Wilcox, LA, Yamada, TK, & Mullin, KD. (2021). A new species of baleen whale (Balaenoptera) from the Gulf of Mexico, with a review of its geographic distribution. *Marine Mammal Science*, 37: 577-610.

Tsai, CH, Fordyce, RE, Chang, CH, & Lin, LK. (2014). Quaternary fossil gray whales from Taiwan. Paleontological Research, 18: 82-93.

Uhen, MD. (2004). Form, function, and anatomy of *Dorudon atrox* (Mammalia, Cetacea): an archaeocete from the middle to late Eocene of Egypt. *University of Michigan Papers on Paleontology* 34:1-222.

Uhen, MD. (2008). A new *Xenorophus*-like odontocete cetacean from the Oligocene of North Carolina and a discussion of the basal odontocete radiation. *Journal of Systematic Palaeontology*, 6: 433-452.

Viglino, M. (2019). Sistemática, filogenia y paleoecología de *Notocetus* *vanbenedeni* del Mioceno temprano de Patagonia y la evolución de los Platanistoidea (Mammalia, Cetacea, Odontoceti). Ph.D. thesis (unpublished). Universidad de Buenos Aires, Ciudad Autónoma de Buenos Aires.

Viglino M, Gaetán CM, Cuitiño JI, & Buono MR. 2021. First toothless platanistoid from the early Miocene of Patagonia: the golden age of diversification of the Odontoceti. *Journal of Mammalian Evolution* *28*: 337-358 DOI 10.1007/s10914-020-09505-w.

Wada, S, Oishi, M, & Yamada, TK. (2003). A newly discovered species of living baleen whale. *Nature*, 426: 278-281.

Yamada, TK, Chou, LS, Chantrapornsyl, S, Adulyanukosol, K, Chakravarti, SK, Oishi, M, ... &

Kurihara, N. (2006). Middle-sized balaenopterid whale specimens (Cetacea: Balaenopteridae) preserved at several institutions in Taiwan, Thailand, and India. *Memoirs of the National Science Museum, Tokyo,* 44: 1-10.
