# Supplementary material for: Breaking the mold: telescoping drives the evolution of more integrated and heterogeneous skulls in cetaceans
Source: PeerJ. 2022 May 5;10:e13392. doi: 10.7717/peerj.13392 (PMC9080436; doi:10.7717/peerj.13392)
Supplement: Supplemental Information 4 [file peerj-10-13392-s004.docx]

**Supplemental figures**

**Breaking the mold: telescoping drives the evolution of more integrated and heterogeneous skulls in cetaceans**

Mónica R. Buono & Evangelos Vlachos


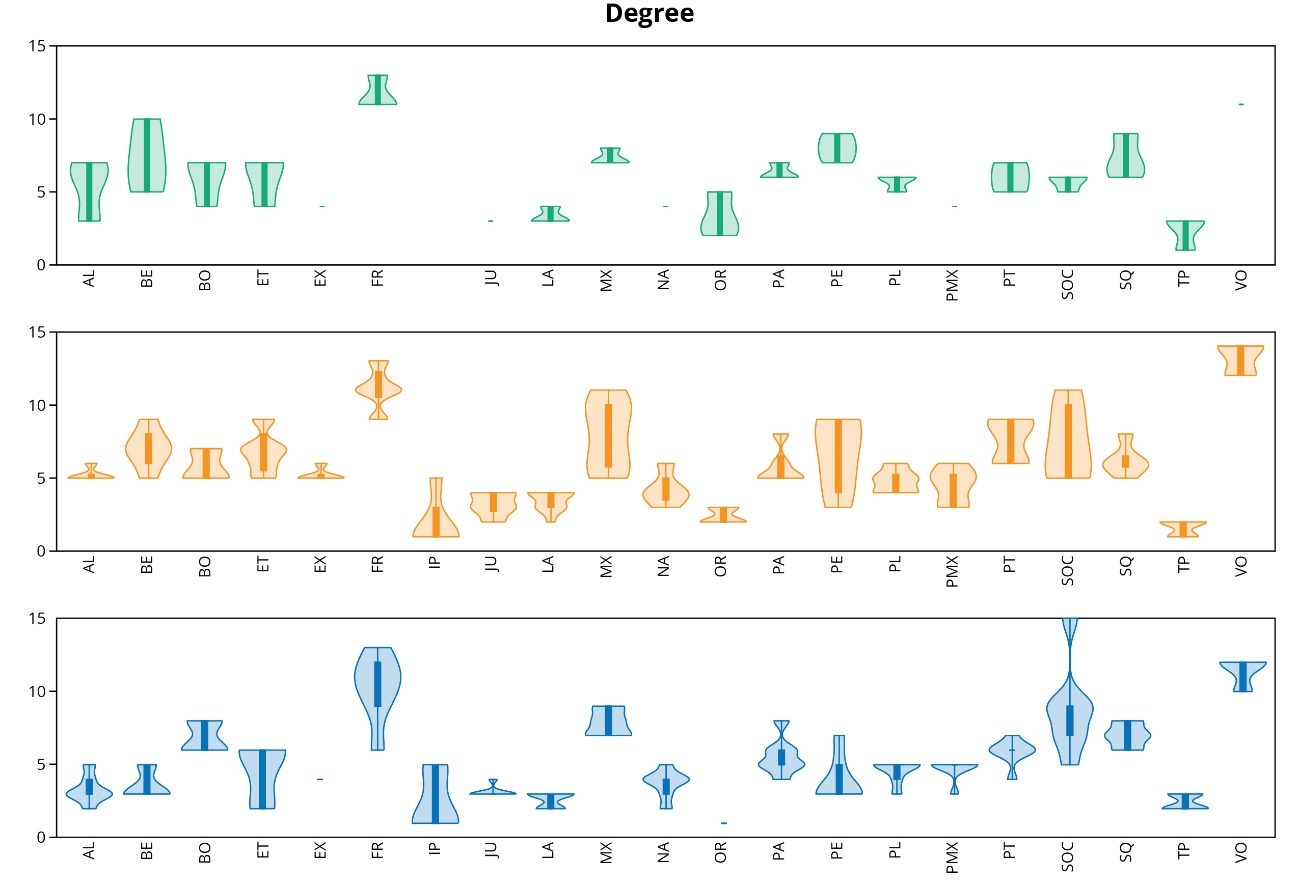


**Figure S1.** Violin plots of the degree of the cranial bones in stem cetaceans (green), odontocetes (orange), and mysticetes (blue). *Balaenoptera* spp. include only *B. acutorostrata* and *B. musculus*. Abbreviations: AL alisphenoid, BE basisphenoid, BO, basioccipital, ET, ethmoid, EX, exoccipital; FR, frontals; IP, interparietal, JU, jugal, LA, lacrimal; MX, maxilla; NA, nasals; OR, orbitosphenoid; PA, parietal; PE: presphenoid; PL, palatine; PMX, premaxilla; PT, pterygoid; SOC, supraoccipital; SQ, squamosal; TP, tympano-periotic; VO, vomer.


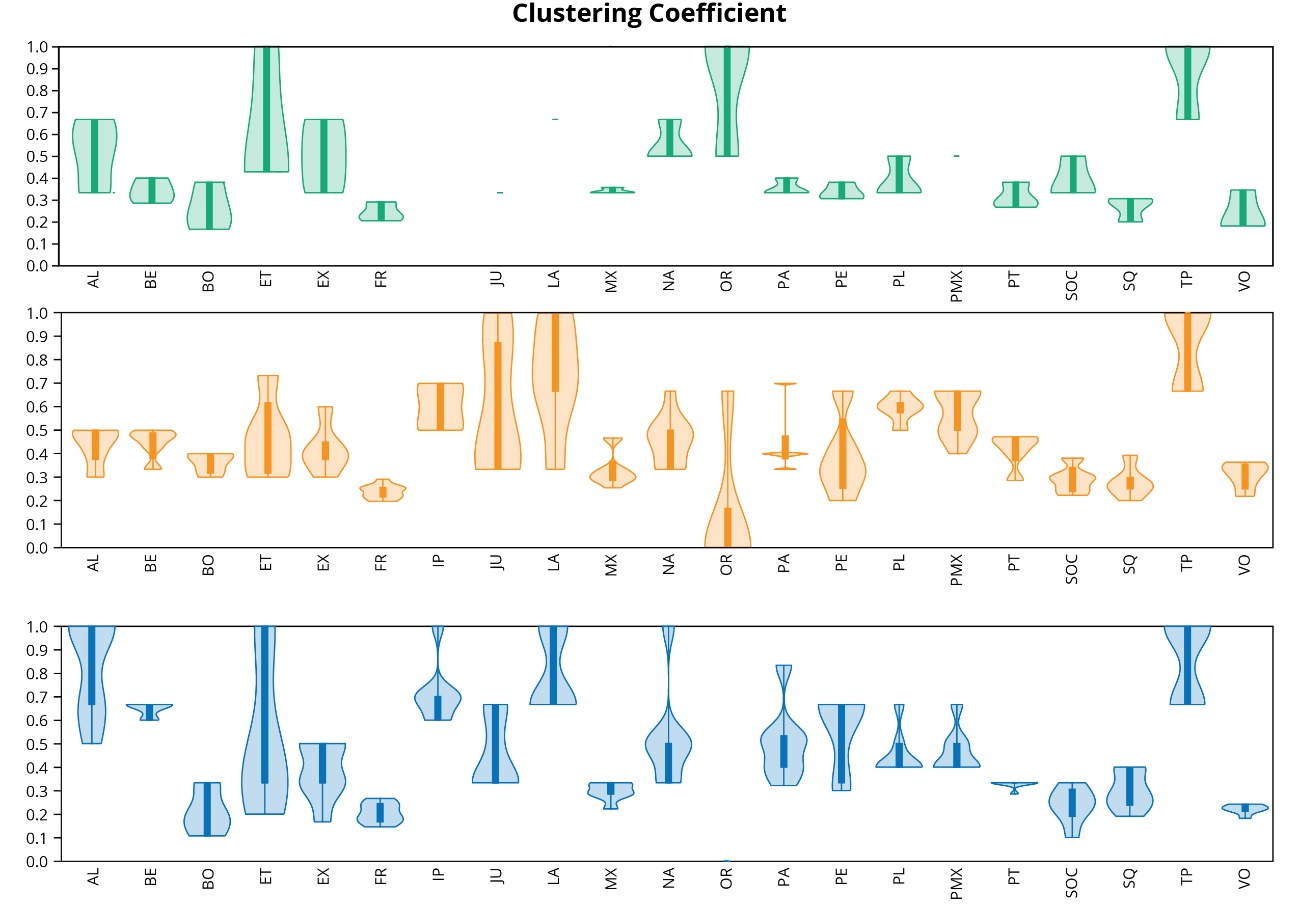


**Figure S2.** Violin plots of the Clustering Coefficient of the cranial bones in stem cetaceans (green), odontocetes (orange), and mysticetes (blue). See bone abbreviations in figure S1.


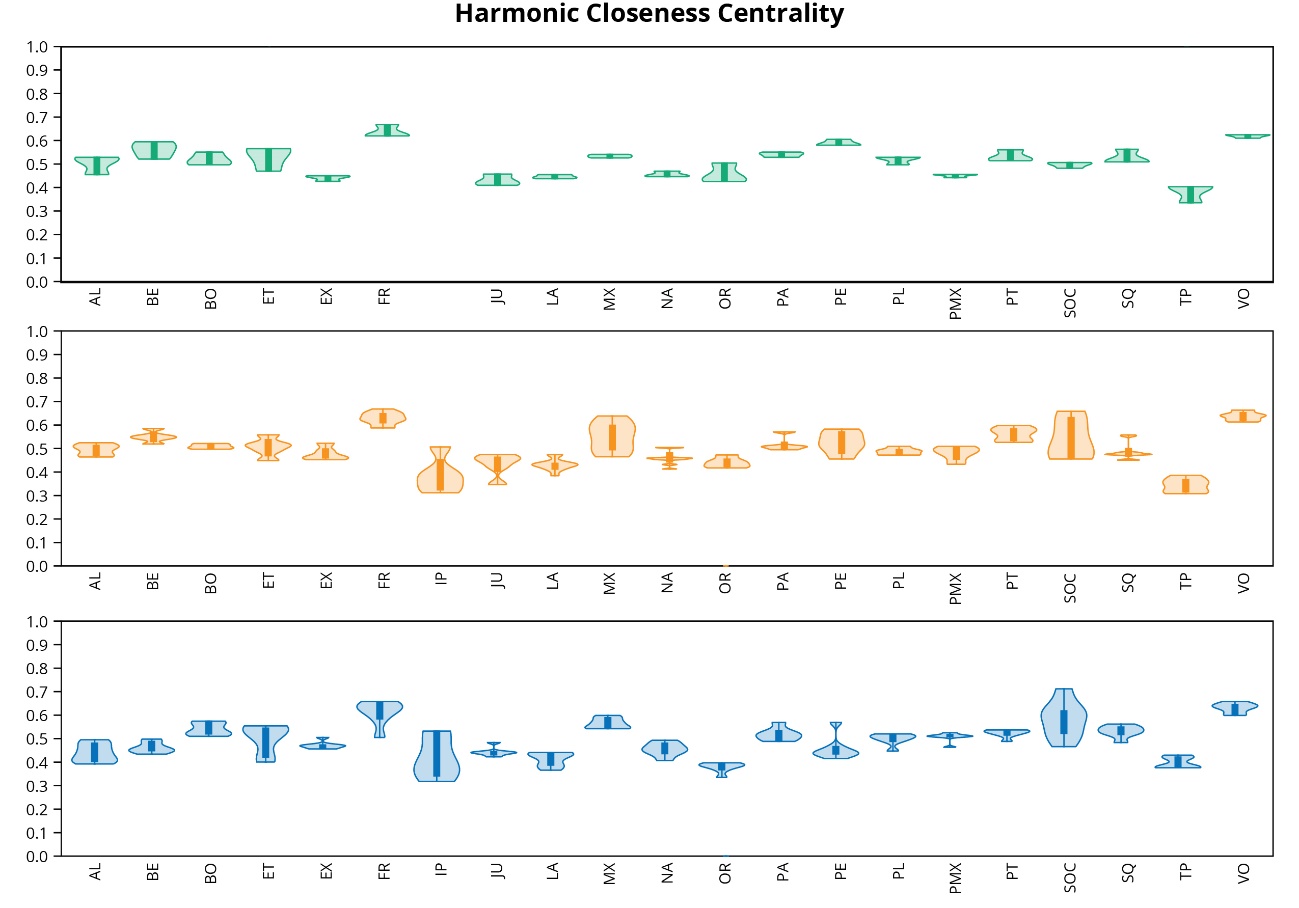
**Figure S3.** Violin plots of the Harmonic Closeness Centrality of the cranial bones in stem cetaceans (green), odontocetes (orange), and mysticetes (blue). See bone abbreviations in figure S1.


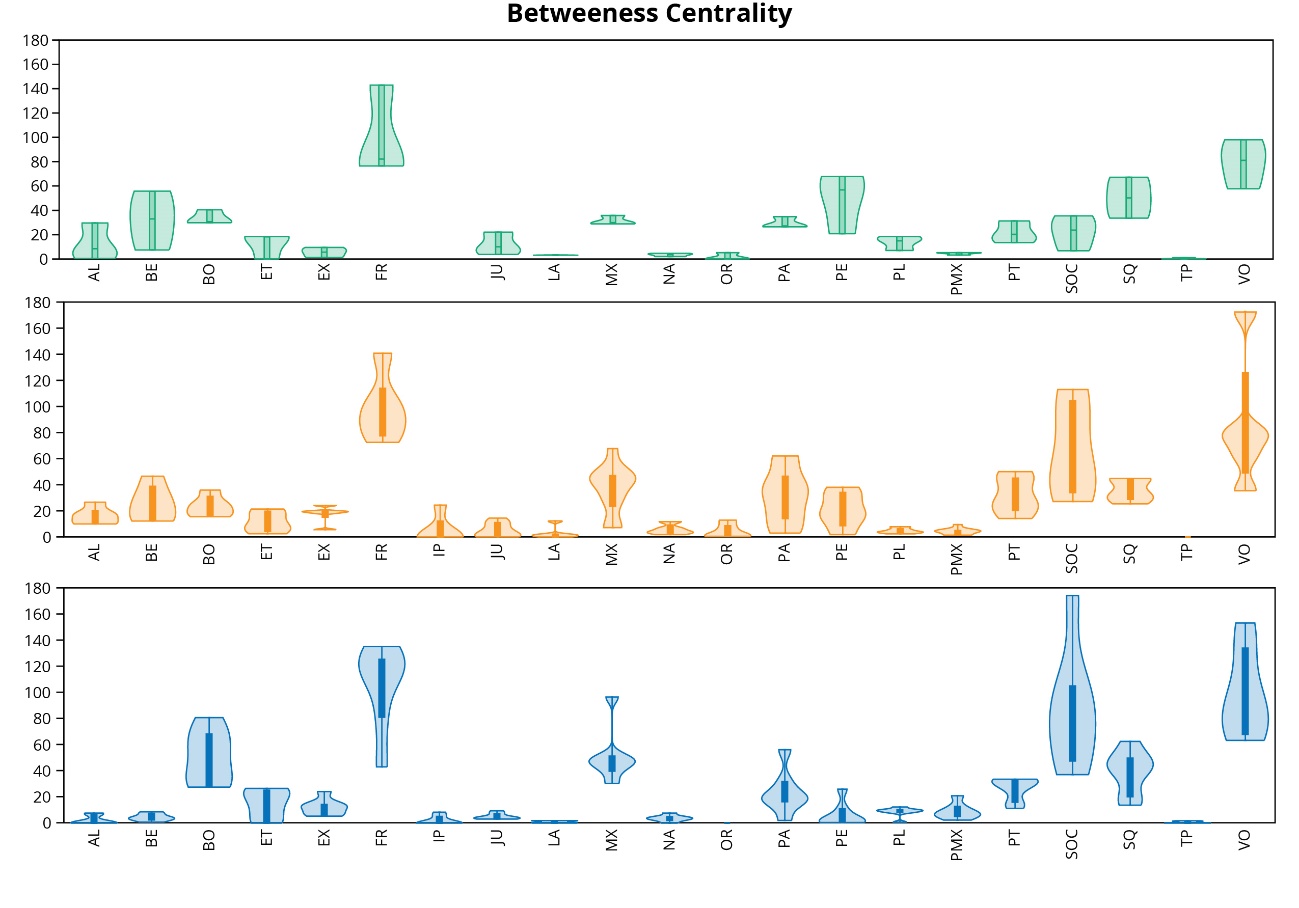


**Figure S4.** Violin plots of the Betweeness Centrality of the cranial bones in stem cetaceans (green), odontocetes (orange), and mysticetes (blue). See bone abbreviations in figure S1.

**Mapping of network properties under maximum parsimony**


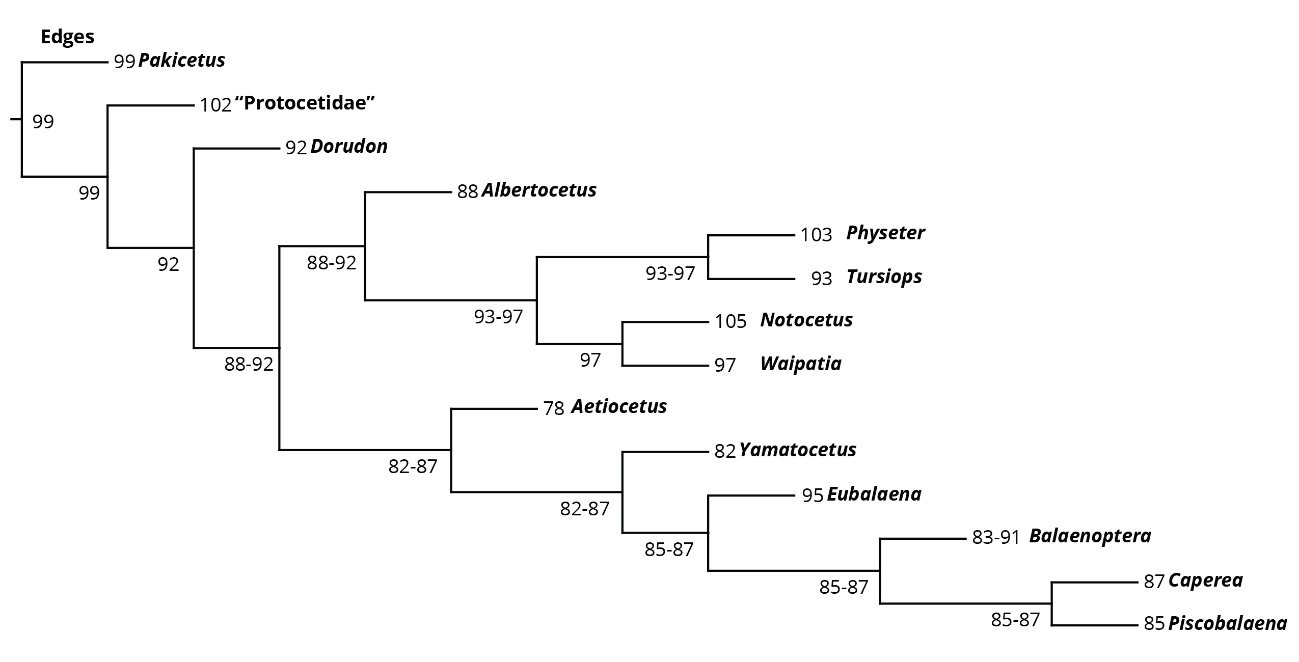


**Figure S5.** Mapping of the evolution of the number of connections among the bones of the cetacean skulls under maximum parsimony, based on simplified phylogeny of Cetacea. An important reduction in the number of connections is noted in Mysticeti. Ancestral reconstructions in and within Mysticeti remain roughly similar, whereas in crown Odontoceti the ancestral reconstructions increase again.


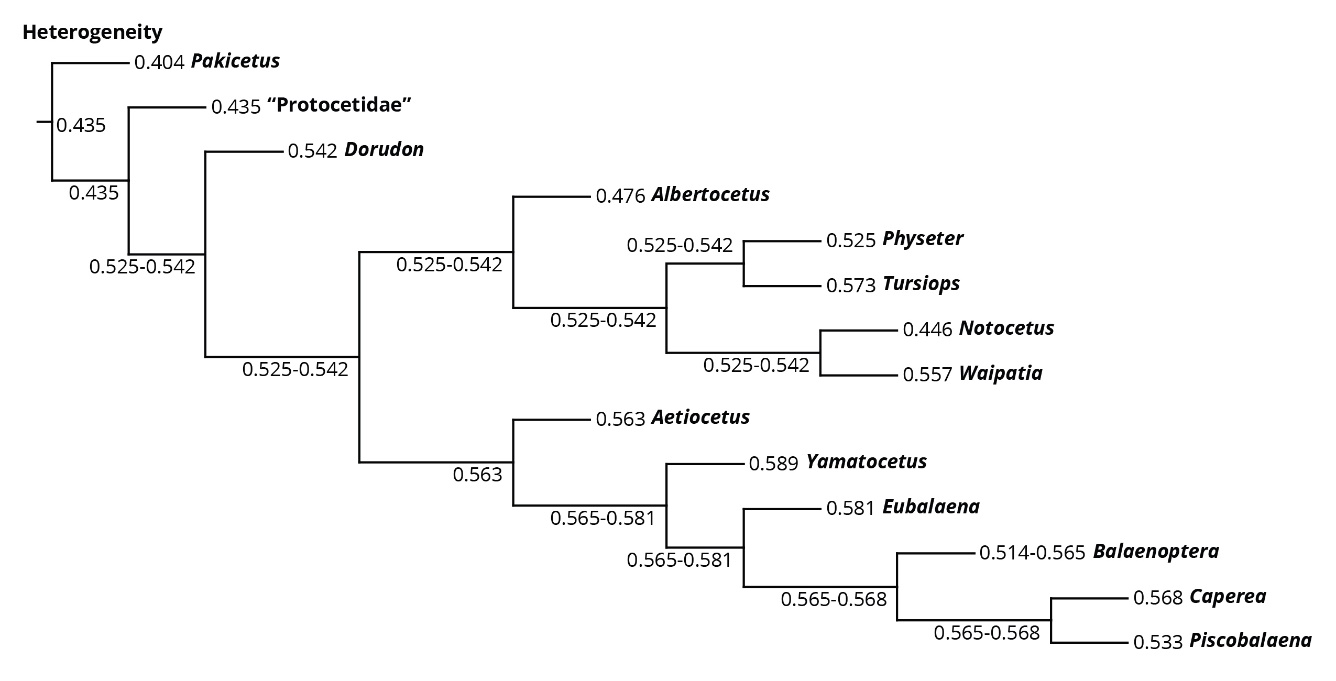


**Figure S6.** Mapping of the evolution of the Heterogeneity of the cetacean skulls under maximum parsimony, based on simplified phylogeny of Cetacea. The ancestral reconstruction of heterogeneity increases in Pelagiceti, to a value that remains stable along Odontoceti. The ancestral reconstructions of heterogeneity further increase along Mysticeti.


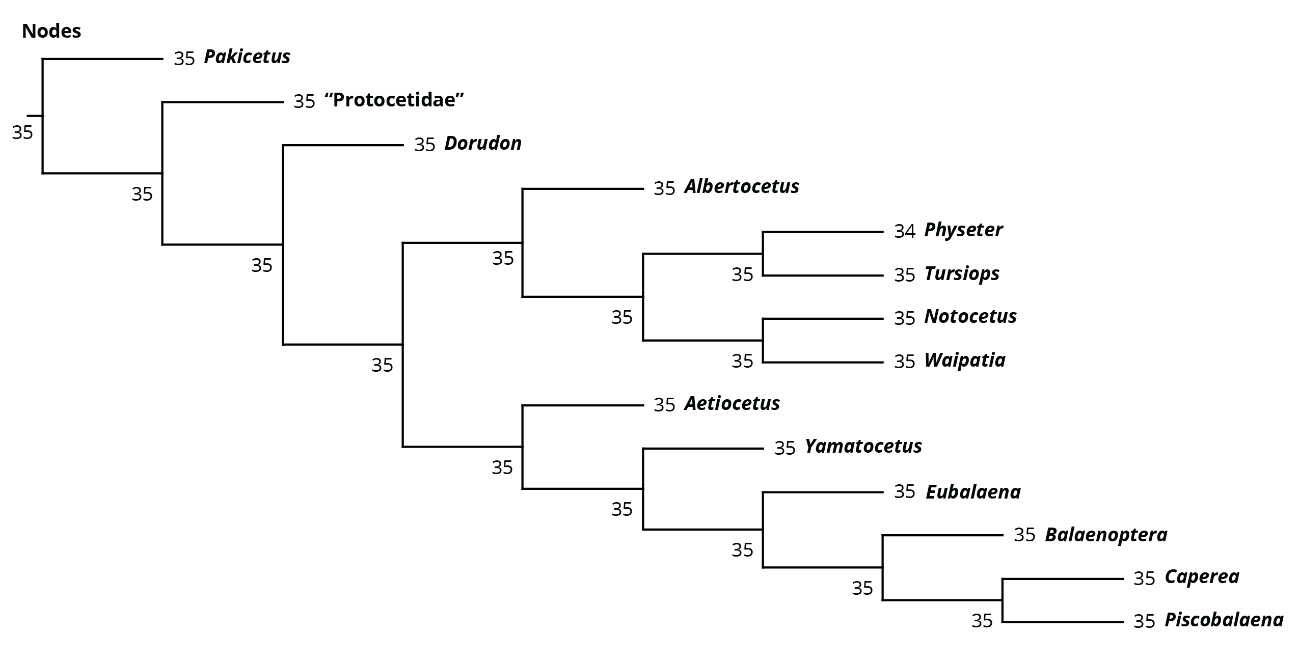


**Figure S7.** Mapping of the evolution of the number of bones in cetacean skulls under maximum parsimony, based on simplified phylogeny of Cetacea. No changes are observed in the ancestral reconstructions during the evolutionary history of cetaceans, and any changes (like the reduction in *Physeter*) are interpreted under parsimony as autapomorphic changes.


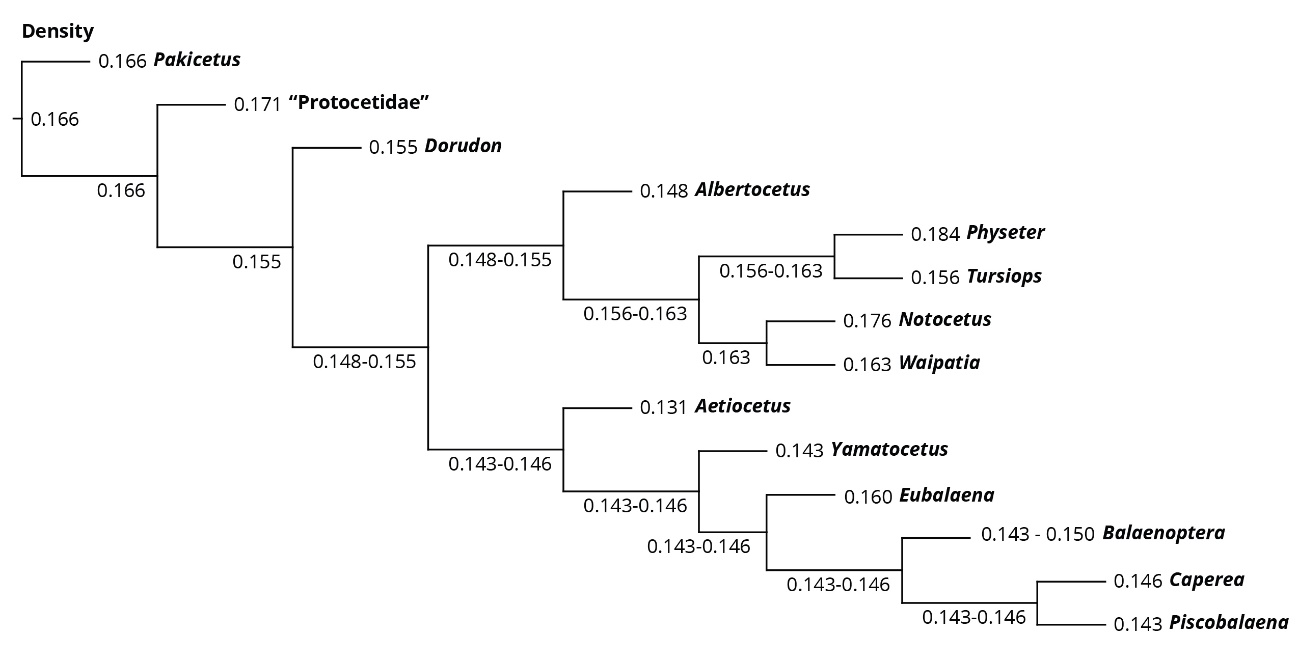


**Figure S8.** Mapping of the evolution of the Density of cetacean skulls under maximum parsimony, based on simplified phylogeny of Cetacea. The ancestral reconstruction of density takes a tendency towards a decline in Neoceti, which is further confirmed in Mysticeti. On the other hand, skulls of crown Odontoceti show an important increase in the ancestral reconstructions of their densities.


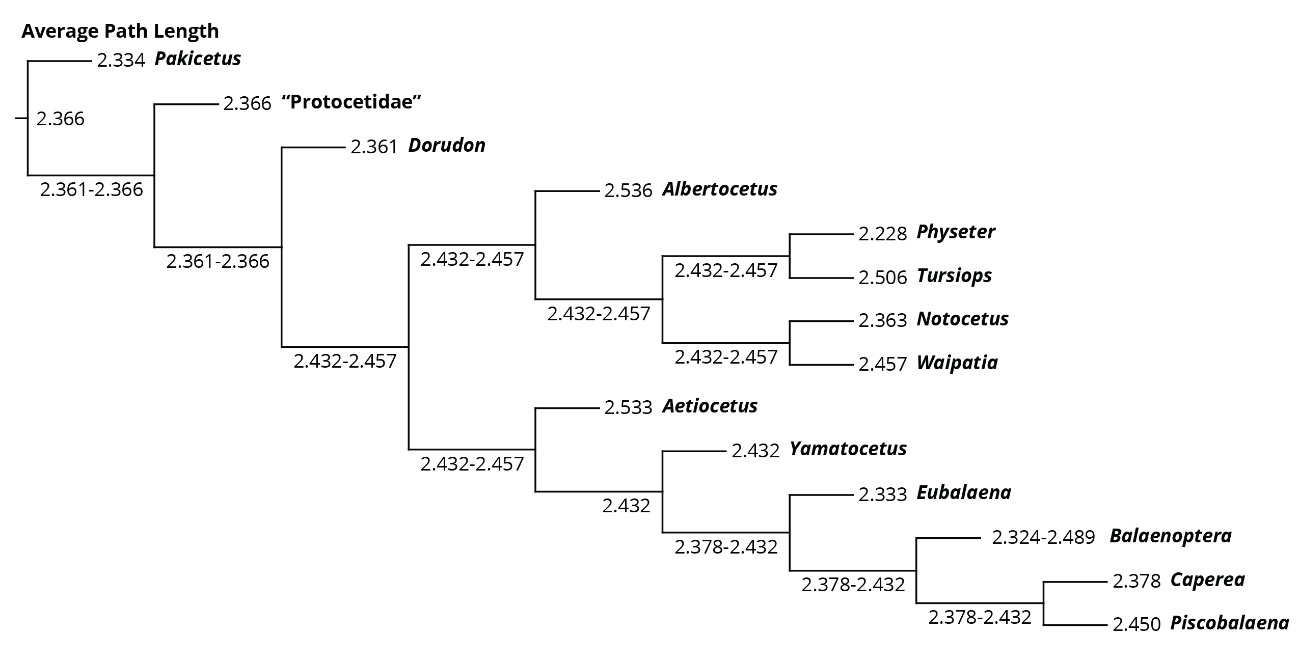
**Figure S9.** Mapping of the evolution of the Average Path Length of the cetacean skulls under maximum parsimony, based on simplified phylogeny of Cetacea. The ancestral reconstruction of the average path length increases in Neoceti (decreasing the integration), a trend that remains stable along Odontoceti and most Mysticeti; in more derived mysticetes, the upper range of the ancestral reconstructions of the average path length slightly decreases (increasing the integration).


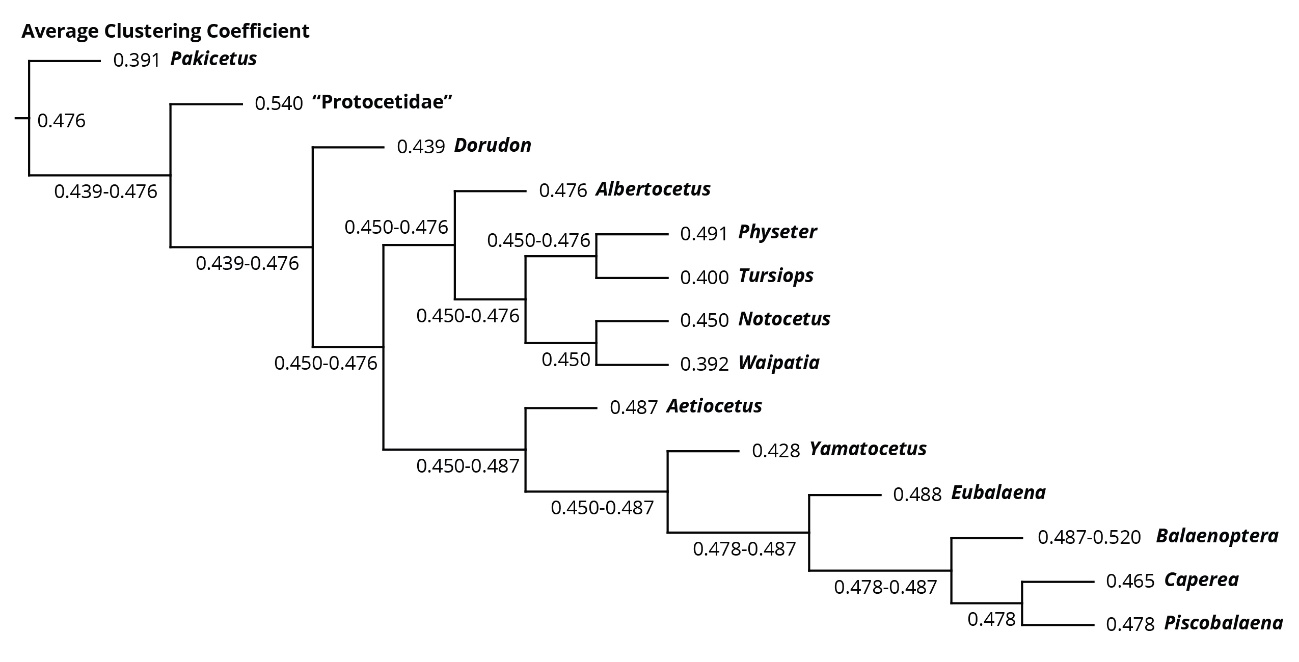


**Figure S10.** Mapping of the evolution of the Average Clustering Coefficient of the cetacean skulls under maximum parsimony, based on simplified phylogeny of Cetacea. The ancestral reconstruction of clustering gradually increases along the basal parts of the tree to take a high value range in Neoceti. Some derived odontocetes show ancestral reconstructions of lower values of this range, whereas along mysticetes clustering slightly increases, at least as far as the maximum range is concerned.


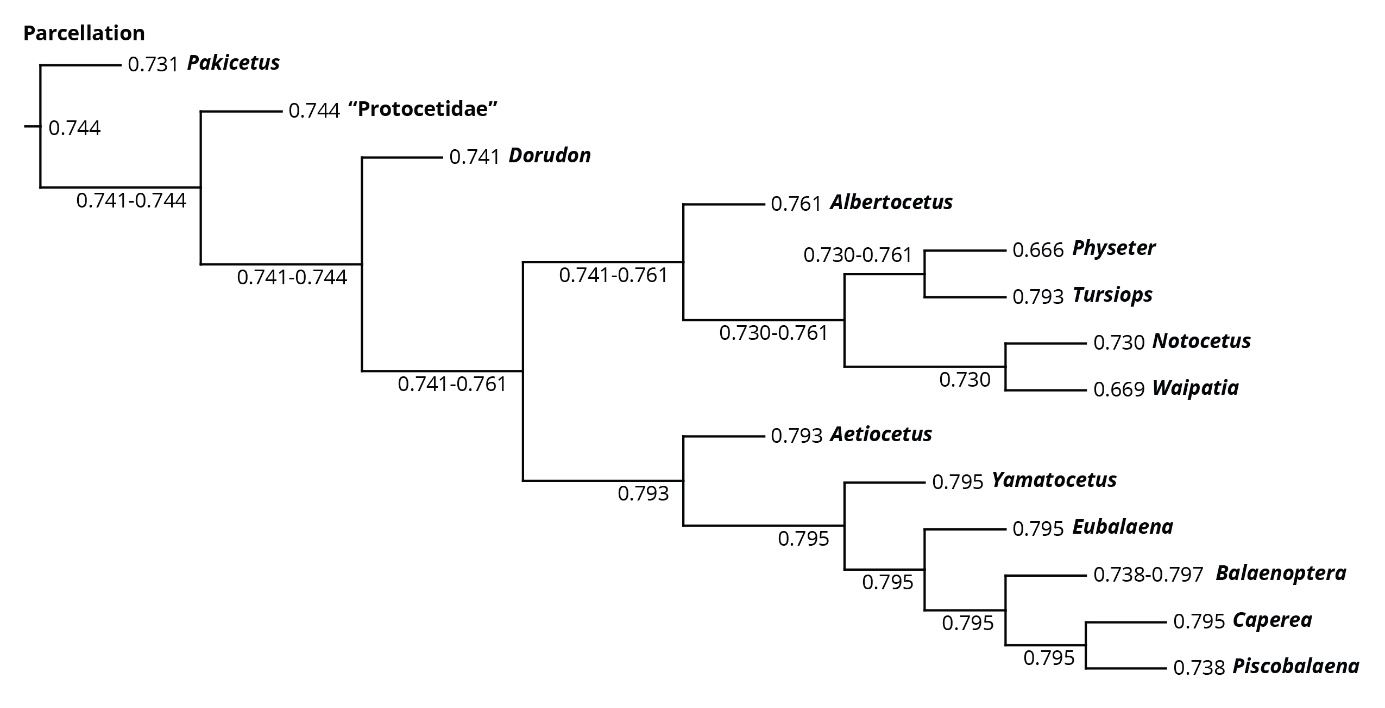


**Figure S11.** Mapping of the evolution of the Parcellation of the cetacean skulls under maximum parsimony, based on simplified phylogeny of Cetacea. The ancestral reconstructions of parcellation change little along the early evolutionary history of cetaceans and Odontoceti, whereas in Mysticeti the overall ancestral reconstructions increase.

**
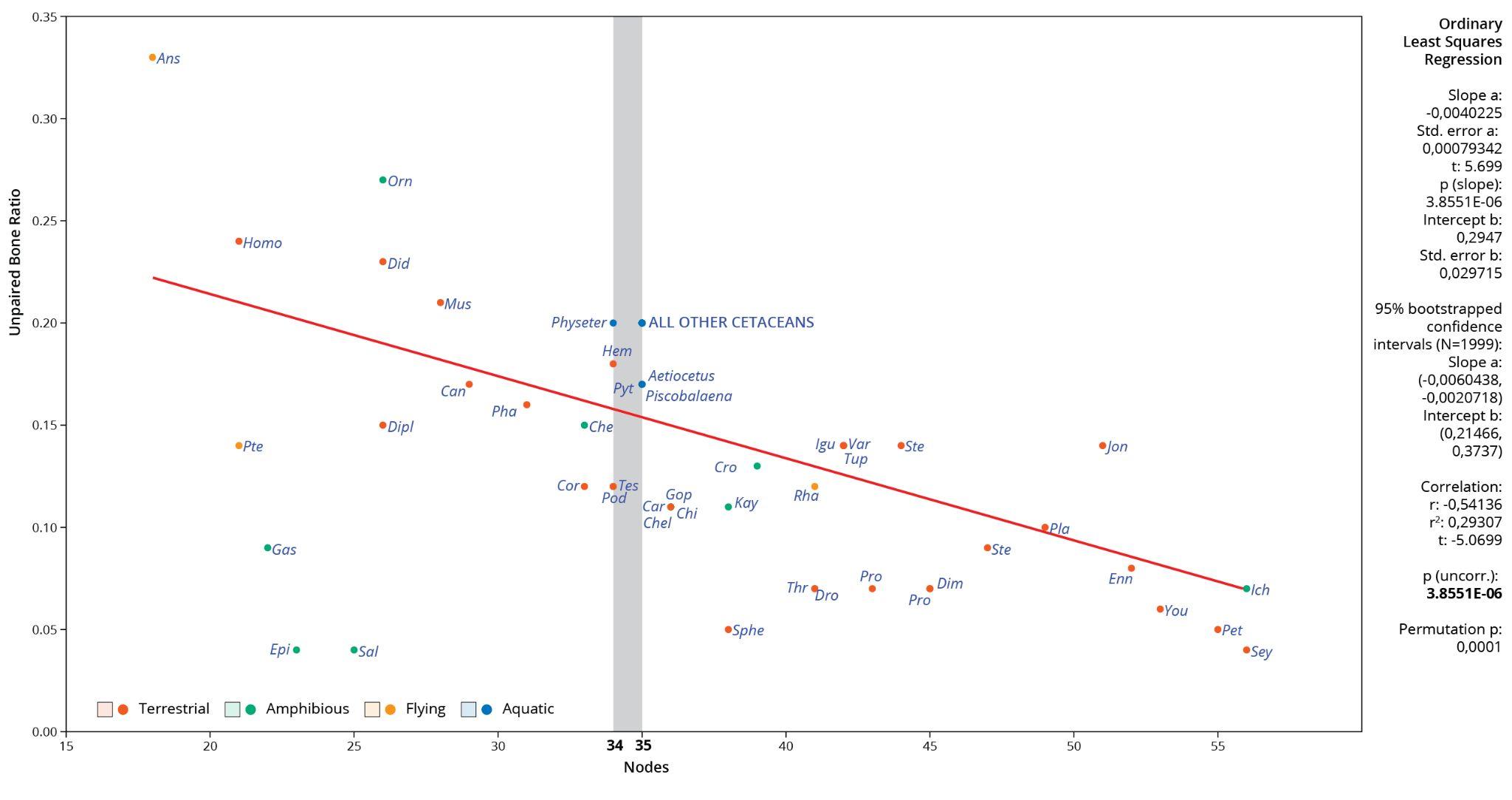
**

**Figure S12.** Correlation between the number of bones and the unpaired bone ratio (UBR) of the tetrapod sample, including the increased cetacean sampling. Among those tetrapods with skulls that contain between 34–35 bones (grey area), cetaceans show the highest UBR, neared only by the snake *Python* and the gecko *Hemitheconyx*. The UBR of cetaceans is also comparable to other derived mammals with fewer cranial elements, like *Homo*, *Mus*, and *Didelphis*. Abbreviations: *Ans*, *Anser*; *Can*, *Canis*; *Car*, *Carettochelys*; *Che*, *Chelodina*; *Chel*, *Chelydra*; *Chi*, *Chisternon*; *Cor*, *Corythosaurus*; *Cro*, *Crocodylus*; *Did*, *Didelphis*; *Dim*, *Dimetrodon*; *Dipl*, *Diplometopon*; *Dro*, *Dromaeosaurus*; *Enn*, *Ennantosaurus*; *Epi*, *Epicrionops*; *Gas*, *Gastrotheca*; *Gop*, *Gopherus*; *Hem*, *Hemitheconyx*; *Ich*, *Ichthyostega*; *Igu*, *Iguana*; *Jon*, *Jonkeria*; *Kay*, *Kayentachelys*; *Orn*, *Ornithorhynchus*; *Pet*, *Petrolacosaurus*; *Pha*, *Phascolarctos*; *Pla*, *Plateosaurus*; *Pod*, *Podocnemis*; *Pro*, *Procolophon*; *Prog*, *Proganochelys*; *Pte*, *Pteropus*; *Pyt*, *Python*; *Rha*, *Rhamphorhynchus*; *Sal*, *Salamandra*; *Sey*, *Seymouria*; *Sphe*, *Sphenodon*; *Ste*, *Stegosaurus*; *Sten*, *Stenocercus*; *Tes*, *Testudo*; *Thr*, *Thrinaxodon*; *Tup*, *Tupinambis*; *Var*, *Varanus*; *You*, *Younginia*.
